# hZIP1 zinc uptake transporter down regulation and zinc depletion in prostate cancer

**DOI:** 10.1186/1476-4598-4-32

**Published:** 2005-09-09

**Authors:** Renty B Franklin, Pei Feng, B Milon, Mohamed M Desouki, Keshav K Singh, André Kajdacsy-Balla, Omar Bagasra, Leslie C Costello

**Affiliations:** 1 Department of Biomedical Sciences, Dental School. University of Maryland, Baltimore, Md, USA; 2 Department of Cancer Genetics, Roswell Park Cancer Institute, Buffalo, NY, USA; 3 Department of Pathology, University of Illinois, Chicago, IL, USA; 4 Department of Biology; South Carolina Center for Biotechnology; Claflin University, Orangeburg, SC, USA

**Keywords:** prostate cancer, zinc, ZIP1 zinc transporter, citrate, ZIP1 gene expression

## Abstract

**Background:**

The genetic and molecular mechanisms responsible for and associated with the development and progression of prostate malignancy are largely unidentified. The peripheral zone is the major region of the human prostate gland where malignancy develops. The normal peripheral zone glandular epithelium has the unique function of accumulating high levels of zinc. In contrast, the ability to accumulate zinc is lost in the malignant cells. The lost ability of the neoplastic epithelial cells to accumulate zinc is a consistent factor in their development of malignancy. Recent studies identified ZIP1 (SLC39A1) as an important zinc transporter involved in zinc accumulation in prostate cells. Therefore, we investigated the possibility that down-regulation of *hZIP1 *gene expression might be involved in the inability of malignant prostate cells to accumulate zinc. To address this issue, the expression of *hZIP1 *and the depletion of zinc in malignant versus non-malignant prostate glands of prostate cancer tissue sections were analyzed. *hZIP1 *expression was also determined in malignant prostate cell lines.

**Results:**

*hZIP1 *gene expression, ZIP1 transporter protein, and cellular zinc were prominent in normal peripheral zone glandular epithelium and in benign hyperplastic glands (also zinc accumulating glands). In contrast, *hZIP1 *gene expression and transporter protein were markedly down-regulated and zinc was depleted in adenocarcinomatous glands and in prostate intra-epithelial neoplastic foci (PIN). These changes occur early in malignancy and are sustained during its progression in the peripheral zone. h*ZIP1 *is also expressed in the malignant cell lines LNCaP, PC-3, DU-145; and in the nonmalignant cell lines HPr-1 and BPH-1.

**Conclusion:**

The studies clearly establish that *hZIP1 *gene expression is down regulated and zinc is depleted in adenocarcinomatous glands. The fact that all the malignant cell lines express *hZIP1 *indicates that the down-regulation in adenocarcinomatous glands is likely due to in situ gene silencing. These observations, coupled with the numerous and consistent reports of loss of zinc accumulation in malignant cells in prostate cancer, lead to the plausible proposal that down regulation of *hZIP1 *is a critical early event in the development prostate cancer.

## Background

Despite the extensive clinical and experimental studies over the recent decades, the pathogenesis of prostate cancer remains unknown. The genetic and molecular mechanisms responsible for and associated with the development of malignant prostate cells and their progression are largely unidentified [for reviews see [1,2]]. The major site for the development of prostate malignancy is the peripheral zone, which comprises about 70% of the prostate gland. It is well established that the normal peripheral zone has the function of accumulating extremely high zinc levels that are 3–10-fold greater than found in other soft tissues [3]. This capability resides in the highly specialized glandular secretory epithelial cells of the peripheral zone, which we characterize as "zinc-accumulating" cells. In contrast, the malignant prostate cells that develop in the peripheral zone do not contain the high zinc levels that characterize the normal secretory epithelial cells. Repeated studies consistently show that the zinc levels of malignant prostate tissue are 62–75% lower than the normal prostate tissue [4-8]. Measurements of pure malignant tissue in the absence of normal glandular epithelium would reveal even lower zinc levels that would approximate the levels found in other soft tissues. This consistency persists in different reports by different investigators employing different populations and tissue samples and involving various stages of malignancy. The studies of Zaichick et al [9] and Vartsky et al [10] further reveal the critically important relationship that, in individual analyses, malignant prostate tissue never exhibits high zinc levels. In addition, Habib [11] reported that the decrease in zinc occurs early in malignancy. These persistent results, and the additional corroborating evidence presented below, firmly establish that the unique zinc-accumulating capability of the normal peripheral zone secretory epithelial cells is lost in the neoplastic transformation to malignant cells; and that zinc-accumulating malignant cells do not exist in situ in prostate cancer. For extensive presentations of the relationships of zinc in normal prostate and prostate cancer, we refer the reader to our recent reviews [12-14].

Established clinical and experimental evidence provides the basis for our concept that zinc accumulation prevents the malignant activities of the neoplastic prostate cell; and that impaired zinc accumulation is an essential requirement for the manifestation of prostate malignancy. If such is the case, one should expect that the zinc-accumulating process that characterizes the normal glandular epithelium is absent or defective in the malignant cells. Until recently, no information had been available regarding the mechanism(s) of zinc accumulation in prostate cells. Recent studies [15-17] have established that the zinc uptake transporter, ZIP1, is important in the uptake and accumulation of zinc by prostate cells. Up-regulation of ZIP1 in prostate cells increases zinc accumulation; and, correspondingly, down-regulation of ZIP1 decreases zinc accumulation in prostate cells. In addition, Rishi et al [18] reported that ZIP1 (and ZIP2) expression in peripheral zone glandular epithelium of black males is down regulated when compared to its expression in white males; which coincides with the race-associated higher incidence of prostate cancer in African-Americans. These relationships suggested that the decrease in zinc in malignant prostate glands might be due to the down regulation of ZIP1 expression. In this report we show, for the first time, the down regulation of h*ZIP*1 gene expression, the loss of ZIP1 transporter protein and the depletion of zinc that is evident in malignant prostate glands. The evidence presented supports the likelihood that down regulation of *ZIP1 *gene expression in the neoplastic prostate cell is an essential step in the development of prostate malignancy. The studies were conducted independently at three different institutions, which strengthens the validity of these corroborating results.

## Results

The studies presented in this report were conducted at the University of Maryland (UMaryland study), the Roswell Park Cancer Institute (Roswell Park Study), and Claflin University (ClaflinU study). Therefore the results will be presented as provided by each separate and independent study, followed by the discussion of the evidence and supporting basis for the genetic/metabolic concept of the role of zinc in prostate malignancy.

### The UMaryland Study (RBF, PF, BM, LCC)

**Figure 1:**
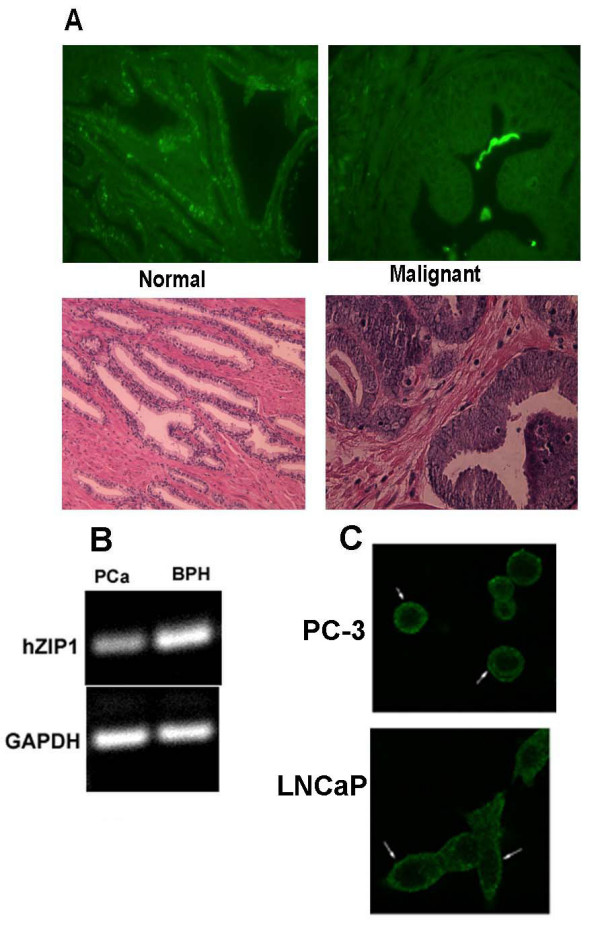
**(A) **Immunohistochemical determination of ZIP 1 transporter levels in normal and malignant prostate glands. The strong positive reaction is evident in the normal gland secretory epithelial cells that border the lumen, and is virtually absent in the malignant glands. Note that ZIP1 is not apparent in the stroma. **(B) **RT-PCR of RNA extracted from malignant prostate tissue and benign prostatic hyperplasia. Note the marked decrease in ZIP1 mRNA in the malignant tissue. Results are representative of two independent samples. Density of the bands was determined by densitometry scans and GAPDH band intensity used to normalize hZIP1 mRNA. hZIP1/GAPDH for PCa and BPH were 0.71 ± 0.067 and 1.02 ± 0.092 respectively. **(C) **Immunohistochemical detection of ZIP1 in malignant prostate cell lines. Note the association of ZIP1 with the plasma membrane.

Earlier studies [15-17] demonstrated that ZIP1 is expressed in malignant prostate cell lines (PC-3 and LNCaP cells); and that this zinc uptake transporter functions in the uptake and cellular accumulation of zinc. This caused us to initiate preliminary studies to determine if *ZIP1 *gene expression and/or the level of the transporter protein might be down-regulated in malignant prostate glands in comparison to the expression in normal prostate glandular epithelium. Paraffin mounted serial sections of human prostate tissue were used for ZIP1 immumohistochemistry staining. Hematoxylin and eosin staining was used for pathologic evaluation of normal glands and adencarcinomatous foci. Figure 1A reveals the membrane-associated immunohistochemical identification of ZIP1 in the normal peripheral zone glandular epithelium. In contrast, the malignant glands were essentially devoid of demonstrable membrane-associated ZIP1. It is also apparent that ZIP1 is confined to glandular epithelium and is not demonstrable in the stromal tissue. Figure 1B presents RT-PCR analysis of ZIP1 expression in tissue extracts of malignant tissue versus benign hyperplastic (BPH) glands; which, like normal peripheral zone, are zinc-accumulating glands. The results demonstrate a relatively high level of ZIP1 gene expression in BPH glandular tissue as compared with a barely detectable expression level in malignant tissue. These results provided the initial preliminary evidence that indicated that down regulation of ZIP1 expression is associated with malignant prostate tissue.

We previously reported the identification by Western blot of the presence of ZIP1 in PC-3 and LNCaP cells under standard culture conditions. These are malignant cell lines that were derived from metastatic prostate tissue. For correlation with the human tissue results, we proceeded to determine the presence of ZIP1 transporter in these cells by immunocytochemistry. Figure 1C shows the localization of ZIP1 in the plasma membrane; which is similar to the localization in normal peripheral zone glandular epithelium. The retention of this gene expression in LNCaP, PC-3, and DU145 (not shown) cells demonstrates that the absence of ZIP1 expression in the malignant glands in situ is not due to the deletion or fatal mutation of the gene. No information exists regarding ZIP1 in metastatic cells in situ in prostate cancer. However, it seems most improbable that the gene would re-appear in metastasis, unless it was reversibly down-regulated in the primary site malignant glands. Therefore the results strongly implicate the epigenetic silencing of *hZIP1 *gene expression in the primary site malignant cells under the in situ environmental conditions of the malignant prostate gland.

These initial observations dictated the importance of expanding the clinical investigation to establish conclusively that ZIP1 is down regulated in prostate malignancy and is associated with a decrease in zinc accumulation in the malignant cells. To achieve this, independent studies were conducted at Roswell Park Cancer Institute and at Claflin University without prior knowledge of the results of the UMaryland study.

### 2. The Roswell Park Study (MMD, KKS)

The Roswell Park (RPCI) resources provided the opportunity to conduct ZIP1 immunohistochemical analysis of prostatic adenocarcinoma slides without identification related to patients. Twenty-two cases of prostatic adenocarcinoma were obtained from RPCI that contained both adenocarcinomatous foci and adjacent benign prostatic hyperplasia (table 1). Four of the cases contained normal prostatic glands and five cases contained prostatic intra epithelial neoplastic foci (PIN). The tumors were graded according to the World Health Organization grading system [19]. Grade 1 is defined by well differentiated glands with minimal anaplasia in which the nuclei are almost uniform with minimal variation in size and shape, and few detectable nucleoli. Grade 2 is defined by moderately differentiated glands with moderate nuclear anaplasia with many nucleoli. Grade 3 is defined by poorly differentiated or undifferentiated glands showing marked anaplasia in which the nuclei showed marked variation in size and irregular shapes, vesicular, with marked abnormal mitotic figures.

**Table 1:** ZIP1 immuno-positivity of glandular components in tissue sections of confirmed cases of prostate cancer.

Case no.	Grade	ZIP1 IHC score^a^			
	
		Normal	PIN	BPH	Malignant
1	3	+++		+++	Negative
2	3		+	++	+
3	1		Negative	+	Negative
4	2			+	Negative
5	2			Negative	Negative
6	1	Negative		+++	Negative
7	2	++		+	+
8	1			+++	+
9	1	++		+++	Negative
10	2			+++	Negative
11	1			++	Negative
12	2			Negative	Negative
13	1			++	+
14	1			+	Negative
15	1			+	Negative
16	1		Negative	+	Negative
17	2			+++	+
18	1		+	++	+
19	1			+	Negative
20	1			+	Negative
21	2		+	+++	+
22	1			Negative	Negative
NEG ZIP1 IHC				3/22 (14%)	15/22 (68%)*
SCORES > +		3/4	0/6	11/22	0/22*
MEAN SCORE^b^		1.75	0.6	1.68(1.09)	0.32(0.48)*

^**a **^Scoring of immunoreactivity was done as follows: negative, no positive cells; score +, <10% positive cells; score ++, 10–50% positive cells; score +++, >50% positive cells

^**b **^MEAN SCORE: MEAN(SD) for each group was obtained by the sum of the +'s/number of cases.

* P < 0.01; BPH VS MALIGNANT GROUPS

**Figure 2:**
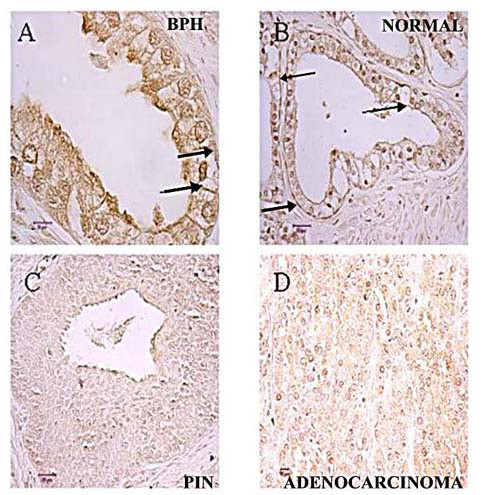
Immunohistochemical detection of ZIP1 transporter protein in malignant and nonmalignant loci of a representative prostate cancer tissue section. (A) BPH, magnification is 1000×, bar = 10 μm. (B) Normal, magnification is 400×, bar = 25 μm. (C) PIN, magnification is 400×, bars = 25 μm. (D) Adenocarcinoma, magnification 400×, bar = 10 μm Note the immuno-positivity of the plasma membrane of BPH and normal glands. The malignant and PIN loci show no detectable ZIP1 so that the plasma membrane of these cells is not visible.

Figure 2 shows the representative results of the ZIP1 immunohistochemical staining observed in normal peripheral zone glands, BPH glands, adenocarcinomatous glands and in PIN. The glandular epithelium of the normal glands and BPH glands (both being zinc-accumulating glands) exhibit immuno-positive ZIP1 staining that is localized predominantly at the basolateral membrane. In contrast, in the adenocarcinomatous glands and PIN, ZIP1 is negligible in the malignant cells so that the appearance of cell membranes is essentially absent. It is also evident that ZIP1 transporter is not detected in the stromal tissue, which corroborates the results of the U.Maryland preliminary study.

**Figure 3:**
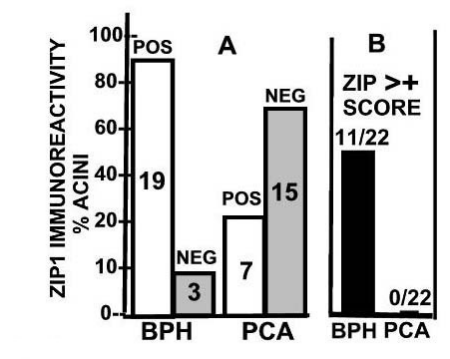
Comparative results of ZIP1 immuno-positive glands of tissue sections from subjects described in Table 1. **A. **Summary of glands that exhibited a positive Zip1 reactivity. The number of cases is shown in each bar. **B**. The number of cases in which the glandular epithelium contained cells that exhibited a ZIP1 score >+ (more than 10% of the cells comprising the acini). The differences in A and B between BPH glands and adenocarcinomatous glands are significant, P < 0.01.

Table 1 is the summary of the immunohistochemical scoring of hZIP1 reactivity of tissue sections from 22 cases of prostate cancer. The analysis involves the comparison of ZIP1 in glands located in the same tissue section. This eliminates, or at least minimizes, any potential technical differences arising from antibody diffusion into the tissue sections and cells for immuno-reactivity. Any comparative differences observed in the immuno-reactivity in the different glands of the same tissue slice would be due to comparative differences in the level of hZIP1. Analysis of the 22 cases (figure 3) for the presence of glands that exhibit ZIP1 immuno-positivity results in a significant difference (P < 0.01) between BPH glands (19 positive/3 negative) and adenocarcinomatous glands (7 positive/15 negative). Analysis for the presence of acini composed of >10% positive cells reveals that BPH glands exhibited this criterion in 50% (11/22) of the cases compared to 0/22 for the adenocarcinmatous glands (figure 3). The average scoring for the twenty-two cases (table 1) was also significantly lower (P < 0.01) for the adenocarcinomatous glands (0.32) as compared to the BPH glands (1.68); i.e. ~5-fold difference. Also, in every case in which the tissue sections showed a positive score for BPH glands, the adenocarcinomatous glands exhibited a lower score. Thus, all the criteria consistently reveal that the immuno-reactive ZIP1 is always reduced and mostly non-detectable in the malignant glandular epithelium. Another important observation is the absence of a correlation between the stage of prostate cancer and the down regulation of ZIP1. This reveals that the down regulation occurs early in the malignant process and persists throughout its progression in the primary site; which is consistent with the early changes in zinc levels.

As would be expected, the presence of normal peripheral zone glands in the malignant tissue sections is minimal, and insufficient for statistical analysis. However in three of the 4 cases, the normal glands exhibited the expected higher ZIP1 expression than the adenocarcinomatous glands, and gave results that were similar to BPH; both of which are zinc accumulating glands. In one case the normal gland was negative for ZIP1, which, seemingly, is an anomaly. However an important point needs to be considered. It is consistent with existing evidence (discussed below) that these metabolic changes occur before the appearance of histopathological evidence of malignant cells. Therefore, this "anomaly" might be due to changes that occur in a "premalignant" neoplastic condition that was histologically identified as "normal". Furthermore, in all five cases with PIN, the glands were either negative or + (none was ++ or +++), which mimics the profile of the adenocarcinomatous glands. It is striking that the combined PIN and adenocarcinoma glands showed no instance of ZIP1 positive cells that exceeded 10%. This could be supportive of a malignant relationship between PIN and adenocarcinoma; but further studies with additional PIN and normal peripheral zone glands are needed. Nevertheless, the Roswell Park study clearly establishes a consistent down-regulation of hZIP1 transporter in malignant prostate glands that corroborates and extends the results of the U.Maryland study, and is further corroborated by the following ClaflinU study.

In a parallel study (unpublished information, to be presented in a separate report), the tissue sections were also assayed for the immunohistochemical identification of m-aconitase. m-Aconitase was prevalent and unchanged in BPH, malignant, PIN and normal glands. Thus the down regulation of ZIP1 is specific. Moreover, the differences in citrate levels in malignant versus non malignant glands is not due to altered levels of m-aconitase. This re-emphasizes the role of altered zinc and ZIP1 in the metabolic transformation associated with prostate malignancy.

### The ClaflinU Study (AK-D, OB)

**Figure 4:**
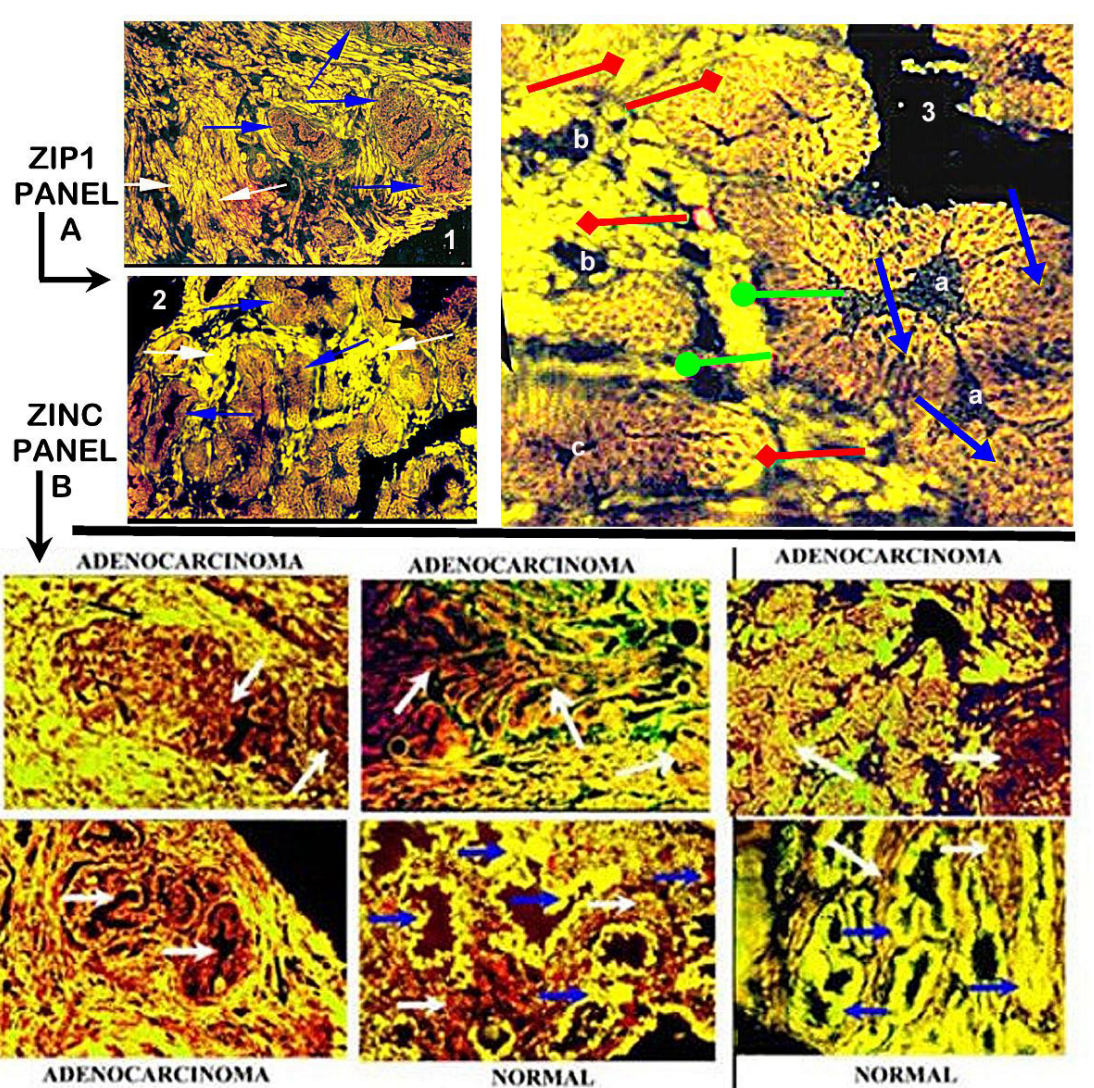
In situ detection of *ZIP1 mRNA *and zinc levels in normal and malignant glands. **Panel A. Representative *ZIP1 *mRNA in Prostate Sections **. Sections (inserts 1,2) from two prostate cancer subjects are shown with low magnification. Blue arrows point to acini with normal glandular epithelium that exhibit ***ZIP1 mRNA***. White arrows point to adenocarcinomatous glands in which ***ZIP1 ***expression is not demonstrable. Insert 3 is a higher magnification of a section from a cancer patient to show more detail. Blue arrows point to acini with normal glandular epithelium. Red arrows point to malignant glands. Green arrows point to stromal (fibromuscular) tissue. The malignant epithelial cells exhibit a complete absence of detectable *ZIP1 *mRNA in the glandular epithelium. The normal glandular epithelium exhibits *ZIP1 *expression; and no *ZIP1 *expression in the stroma. Normal acini marked 'a' show uniform *ZIP1 *mRNA expression in the glandular epithelium. Advanced adenocarcinomatous glands marked as 'b" show uniform absence of *ZIP1 *mRNA. Developing early stage adenocarcinomatous glands marked 'c' show a progression of normal *ZIP1 *expressing cells and malignant cells that lost the expression of *ZIP1*. **Panel B. Representative Zinc Levels in Prostate Sections. **High zinc is represented by Newport Green yellow stain and low zinc is represented by TSQ red stain. The malignant region of the peripheral zone shows a significant depletion of zinc in the malignant glandular epithelium as exhibited by the red staining (white arrows). The depletion of zinc is evident in early differentiated malignant glands as represented by combinations of red and yellow staining in the glandular epithelial cells. As malignancy advances to the undifferentiated stage, the zinc is further depleted as represented by the dominant red stain and no yellow stain in the glandular epithelium of the adenocarcinomatous glands. The depletion of zinc in the malignant glandular region results in the surrounding stroma showing a higher zinc level (green stain) than the glandular epithelium. In contrast, the normal peripheral zone glands exhibit high zinc levels as represented by the uniform yellow stain and absence of red stain in the glandular epithelium. The stroma surrounding the glands exhibits a lower zinc level as shown by the red stain.

In this study, ZIP1 mRNA expression (RT-in situ-PCR) and the relative level of zinc content were determined in the normal peripheral zone glands versus malignant glands from 38 prostate resections. The typical results represented in Figure 4 were consistently observed in all 38 prostate resections. The results show that *hZIP1*gene expression is evident uniformly in the epithelium of the normal peripheral zone glands; and is absent in the stroma. *hZIP1 *expression is markedly down regulated to the extent of not being demonstrable in the adenocarcinomatous glands; and presents the same appearance as the surrounding stroma. Of significance is the apparent down regulation of ZIP1 in early-stage as well as in advanced-stage malignant glands; which is consistent with the decrease in ZIP1 transporter protein shown in the Roswell Park study.

Correspondingly, Figure 4 shows the high level of cellular zinc that characterizes the normal glandular epithelial cells (green color). In contrast, the stroma exhibits a low level of zinc. Therefore, the in situ zinc staining provides the expected differential in zinc between normal glandular epithelium and stroma. The marked reduction of cellular zinc in the epithelium of the adenocarcinomatous glands is apparent. Like the expression of ZIP1, the loss of zinc occurs early in malignancy. Due to the depletion of zinc in the malignant glands, the stromal zinc level gives the appearance of relatively higher zinc levels. Many studies have observed that zinc levels are greatly decreased in extracts of resected malignant tissue preparations. However, the ClaflinU study provides the first in situ detection of the depleted cellular zinc levels in adenocarcinomatous glands as compared to the high zinc levels in normal glandular epithelium. An important revelation is that the decrease in zinc level in the malignant glands is due to a decrease in the cellular accumulation of zinc. This establishes that the decrease in intracellular zinc, and not impaired secretion of zinc into the lumen (prostatic fluid), is principally responsible for the decrease in malignant tissue zinc level. Thus, the results of the ClaflinU study are consistent with and corroborate the Roswell Park study and the preliminary results of the UMaryland study.

## Discussion

### The Zinc-Citrate Connection in Normal and Malignant Prostate

**Figure 5:**
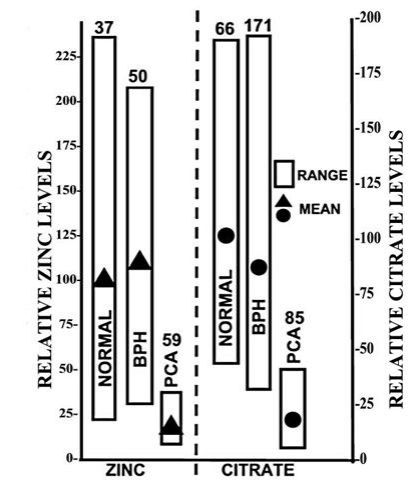
Composite of zinc and citrate levels in prostate. The zinc data are taken from Zaichick et al [9] and show the range of zinc levels in resected prostate tissue samples from different subjects. The citrate data are taken from Kurhanewicz et al [41] and show the range of citrate levels as determined by in situ magnetic resonance spectroscopy imaging of the prostate gland of different subjects. The actual zinc and citrate concentrations for normal were set to 100 and the values for BPH and PCa were adjusted accordingly. Note the parallelisms in that zinc and citrate levels are consistently significantly low in malignancy; and that no case exists in which the malignant loci retain the high zinc or high citrate levels that characterize normal or hypertrophic glands. The values above each bar are the number of subjects.

The results of the present study coupled with the numerous and consistent reports of others [[4-8,12-14] for reviews], provide direct overwhelming clinical and experimental evidence that, in prostate cancer, the lost ability of the malignant cells to accumulate zinc is a consistent event in the development of malignancy. However, the significance and further corroboration of this relationship requires the understanding and recognition of the unique role of zinc in normal prostate function and in prostate cancer. The major function of the human prostate gland peripheral zone (as in other animals) is the production and secretion of enormously high levels of citrate; which we refer to as "net citrate production". This capability of the normal secretory epithelial cells is the result of their unique ability to accumulate high levels of zinc; which inhibit m-aconitase activity and citrate oxidation [20,21]. Thus, one must recognize that the production and accumulation of citrate is dependent upon and is preceded by the accumulation of zinc in the glandular epithelial cells. Therefore, changes in the level of citrate in the peripheral zone are the result of and indicative of changes in zinc levels. The recent development of in situ magnetic resonance spectroscopy of prostate citrate levels in normal peripheral zone and malignant loci conclusively establishes that citrate levels are always greatly reduced in malignancy [see reviews [22-24]]. The consistency of this citrate relationship now makes magnetic resonance spectroscopy imaging (MRSI) of the prostate gland the most effective and reliable procedure for the identification, localization and volume estimation of malignant loci in the peripheral zone. Data collected from virtually all the existing MRSI reported studies reveal that there exists no case in which the malignant loci retain the high citrate levels of the normal peripheral zone glands as represented in figure 5[13,22]. These citrate changes revealed by magnetic resonance spectroscopy provide indirect evidence of corresponding changes in the accumulation of zinc, which is the cause of the changes in citrate. This is further verified by the comparative changes in zinc shown in figure 5. The profile of direct measurements of zinc changes associated with malignant prostate tissue strikingly replicates the citrate profile. This is evident despite the fact that these are different studies with different subjects and different stages of cancer. These relationships provide compelling evidence that, in prostate cancer, the malignant cells lose the ability to accumulate high zinc levels; and malignant cells that retain the accumulation of high zinc levels virtually never exist.

### The Concept of the Role of hZIP1 and Zinc in Prostate Cancer

The existence of the zinc and citrate relationships in normal prostate and prostate cancer is irrefutable. How these relationships are involved as factors in the development and progression of prostate malignancy is important to understanding the pathogenesis of prostate cancer. It is well documented that all tumor cells undergo metabolic transformations that are essential for their malignant existence (25, 26 for reviews). It is important to emphasize that these metabolic transformations are not the cause of malignancy. Malignancy requires the genetic transformation of a sane cell to a neoplastic cell that is endowed with the potential capability of malignancy. The metabolic transformation is essential for the neoplastic cells to manifest their malignant capabilities.

The accumulation of zinc in normal prostate glandular epithelial cells results in two important effects; a metabolic effect and a proliferative effect. Its metabolic effect is the inhibition of citrate oxidation that is essential for the prostate function of production and secretion of high levels of citrate [20,21]; and its inhibition of terminal oxidation [27]. This has a bioenergetic cost in that the inhibition of citrate oxidation results in a ~60% loss of ATP production that would arise from complete glucose oxidation. Consequently, zinc-accumulating citrate-producing cells (normal peripheral zone epithelial cells) are energy-inefficient cells. A second effect of zinc is its inhibition of prostate cell proliferation. This effect results from zinc induction of apoptosis in prostate cells [28-31]. These are the consequences imposed upon highly specialized zinc-accumulating citrate-producing cells (i.e. normal peripheral zone secretory epithelial cells) in order to achieve their unique function of net citrate production.

Malignant prostate cells do not exist for the specialized function of citrate production and secretion. They must replace the metabolic pathways associated with net citrate production with metabolic relationships that are suitable for their malignant existence. That the malignant prostate cells in situ never exist as zinc-accumulating, citrate-producing cells is evidence of the incompatibility of the high zinc accumulation and net citrate production for their existence. Their metabolic transformation to energy-efficient citrate-oxidizing cells that have lost the ability to accumulate zinc provides their metabolic/bioenergetic requirements of malignancy. Also, the apoptotic influence of zinc is eliminated, which permits the proliferation of the malignant cells. However, the evidence presented herein clearly establishes hZIP1 down regulation in the primary in situ site and further suggests that this is the explanation for the consistently observed decrease in zinc levels in prostate cancer.

**Figure 6:**
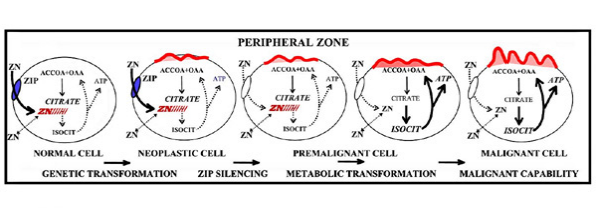
The integrated role of ZIP1, zinc, and citrate metabolism in the pathogenesis of prostate malignancy. The normal glandular epithelial cell expresses ZIP1 that permits zinc accumulation, which inhibits citrate oxidation and terminal respiration. Citrate accumulates and coupled ATP production is reduced. A genetic transformation results in a neoplastic cell with potential malignant capability. *ZIP1 *expression is silenced by epigenetic factors which eliminate Zip1 transporter and accumulation of zinc in the premalignant cell. The level of cellular zinc decreases which removes the inhibitory effects on citrate oxidation and terminal oxidation. The Krebs cycle is functional and coupled ATP production is increased. The malignant cell is metabolically and bioenergetically capable of manifesting its malignant potential. Additionally, the growth inhibitory effect of zinc is removed, which allows growth and progression of the malignant cell.

This concept is represented in figure 6. The occurrence of this metabolic transformation is dependent upon the ability of the normal epithelial cells and the inability of the malignant cells to accumulate zinc. The present studies establish that *hZIP1 *is down-regulated in the adenocarcinomatous glands. This is consistent with the down-regulation of *hZIP1 *gene expression in the African-American male population, which exhibits a higher incidence of prostate cancer [18]. The functional importance of hZIP1 in the accumulation of zinc in prostate cells has been established [15-17]. Over-expression of hZIP1 results in increased accumulation of zinc which leads to inhibition of cell proliferation; whereas cells with down-regulation of hZIP1 have decreased cellular zinc levels and increased proliferation. Also the accumulation of zinc in the malignant prostate cells in culture and in vivo [31] results in increased citrate levels.

Consequently, consistent clinical and experimental evidence strongly implicate the down-regulation of hZIP1 in the lost ability of the malignant cells to accumulate zinc. The existence of hZIP1 insures that prostate cells will accumulate zinc. If ZIP1 is not down regulated in the neoplastic cell, zinc accumulation and its metabolic/energetic and apoptotic effects will prevail; and the neoplastic cell will remain in a pre-malignant dormant state and/or will die. In this concept (figure 6), prostate malignancy requires two essential transformations; the genetic transformation to a neoplastic cell with potential malignant capability; and the metabolic transformation to an energy-efficient citrate-producing cell that has lost the ability to accumulate zinc. These relationships provide a plausible explanation and expectation for the apparent absence of the identification of malignant prostate glands that exhibit high zinc and high citrate levels.

The present studies raise two related important issues that we will be investigating: what is the cause of the down regulation of ZIP1? ; do the ZIP1 and zinc changes persist in the metastatic cells in situ? No information currently exists regarding the latter issue. The fact that hZIP1 is expressed in prostate cancer cell lines (that were established from metastatic lesions) suggests that down regulation of hZIP1 is a reversible phenomenon that occurs in the primary site in situ. This is suggestive of an epigenetic effect imposed by the interaction of the neoplastic cells and their in situ environment. In this case, the in vitro conditions of the cultured cells would eliminate the in situ factor(s) associated with the suppression of hZIP1 expression; thus permitting its re-expression. Moreover, the re-expression in the culture cells results in functional hZIP1 that manifests zinc uptake ; so that a fatal mutation is not involved. It is notable that SLC5A8, a gene that encodes a monocarboxylic acid transporter protein, has been reported to be a tumor suppressor gene in colon cancer [32-35] and other cancers [36,37]. The silencing of that gene occurs by hypermethylation and is a common and early event in human colon cancer. Similarly, it is plausible to propose that hZIP1 is a candidate tumor suppressor gene in prostate cancer. It will be important to determine the in situ conditions and mechanism that initiates the silencing of ZIP1 gene expression; which will then provide an understanding of the etiology of prostate malignancy.

The focus of this report on ZIP1 is not to imply that other zinc transport processes might not be involved in the altered accumulation of zinc. Rishi et al [18] demonstrated that ZIP1 and also ZIP2 are expressed in human prostate glandular epithelium. An increase in export of zinc could also decrease zinc accumulation by "true" malignant cells. However no information currently exists concerning the functional role of zinc exporters in prostate cells. Beck et al [38] reported that ZnT-4 was decreased in peripheral zone malignant tissue when compared to normal peripheral zone tissue samples. ZnT-4 is associated with the sequestering of cytosolic zinc into organelles, and is not involved as a plasma membrane zinc exporter. Moreover, a decrease in ZnT-4 would not be associated with a decrease in cellular zinc level, even as a secretory process. They also reported that ZnT-1 expression was unchanged in malignant versus normal peripheral zone. ZnT-1 does function as a plasma membrane-associated zinc exporter in some cells and possibly in prostate cells. Hasumi et al [39] reported that ZnT-1 expression was significantly lower in malignant prostate tissue samples when compared to BPH samples, which led them to conclude that ZnT-1 was not likely to be associated with the decreased zinc accumulation in the malignant cells. Consequently, a possible role of altered expression of zinc exporters in the genetic/metabolic transformation of the malignant cells in situ is not evident, but more research is required regarding this issue. We are now investigating the possible involvement of other zinc transporters in prostate malignancy.

## Conclusion

The present studies, conducted independently in three institutions, collectively establish the presence of *hZIP1 *gene expression, the presence of membrane-associated hZIP1 transporter protein, and the accumulation of cellular zinc in the normal peripheral zone glandular epithelium and in benign hyperplastic glandular epithelium. The studies reveal that *hZIP1 *gene expression is down-regulated and hZIP1 transporter protein is depleted in adenocarcinomatous glands in prostate cancer. Correspondingly, the cellular level of zinc is also depleted. These effects occur in early and late stages of malignant development of the peripheral zone. *hZIP1 *expression is evident in the malignant prostate cell lines in culture. This leads to the likelihood that the lost expression in the adenocarcinomatous glands is due to an epigenetic silencing of *hZIP1 *that occurs in the in situ environment of the peripheral zone. When coupled with the voluminous clinical and experimental evidence, it becomes irrefutable that the development of malignancy in prostate cancer involves an essential metabolic transformation that results in the lost ability of malignant cells to accumulate zinc. Conversely, as long as the capability of high zinc accumulation exists, the neoplastic cells cannot manifest their malignant potential. Consequently, the expression of *hZIP1 *that sustains zinc accumulation in prostate cells will prevent the malignant activities and proliferation of the neoplastic cells. This provides a compelling basis for the proposal that *hZIP1 *down regulation is necessary for tumor progression and could be a tumor suppressor gene in prostate cancer. Consideration of all the clinical and experimental evidence leads to the concept that zinc and citrate-related metabolism play an important role in the pathogenesis and progression of prostate malignancy.

## Methods

### 1. U.Maryland Study

#### Immunohistochemistry of Human Tissue Sections

Paraffin mounted serial sections of human prostate tissue was used for hZIP1 immunohistochemistry staining. Hematoxylin and eosin staining was used for identification of normal and adenocarcinomatous glands. For immunohistochemistry, slides were dewaxed by incubation in xylene and then rehydrated. Non-specific binding of antibody was blocked by incubation in BlokHen (Aves Labs, Inc.) solution. The slides were washed with PBS, incubated in hZIP1 antibody solution, washed again, and incubated with fluorescein-labeled secondary antibody solution; and then washed and mounted with anti-fade fluorescent medium (Molecular Probes). For control staining, adjacent serial sections were stained as described above except that the antibody-depleted and preimmune preparation were used instead of antihZIP1 antibody

#### Immunocytochemistry of Prostate Cells

PC-3 and LNCaP cells were plated on cover slips. The cover slips were washed with PBS, and the cells fixed in paraformaldehyde solution. The cells were permeabilized by incubation in 0.2% NP-40 solution, washed in PBS, and stained by the procedure described above for immunohistochemistry.

##### RT-PCR of Human Tissue mRNA

hZIP and GAPDH cDNA were synthesized from total mRNA isolated from human prostate tissue using 1.0 ug of total RNA, reverse transcriptase and random primers (TaqMan7 reagents, Perkin Elmer). hZIP1 and GAPDH fragments were amplified from the cDNA using 1.0 μM forward and reverse primers and 35 cycles. These conditions were shown to be in the quantitative detection range based on the concentration of template DNA. The cloned cDNA for hZIP1 was used as the template DNA in control reactions to determine the specificity of the PCR reactions. The RT-PCR products were analyzed by agarose gel electrophoresis with ethidium bromide staining and photographed under UV light. No products were detected without reverse transcriptase. The primers for hZIP1 were 5'-TCAGAGCCTCCAGTGCCTGT-3' and 5'-GCAGCAGGTCCAGGAGACAA-3'

### 2. The Roswell Park Study

#### Immunohistochemistry of Human Tissue Sections

Embedded prostatic adenocarcinoma slides that contained both benign prostatic hyperplasia (BPH) and adenocarcinomatous foci were obtained from Roswell Park Cancer Institute. Normal glands and intra epithelial neoplastic foci (PIN) were seen in a few cases. Immunohistochemistry with anti-hZIP1 antibody was performed by standard protocol [40]. Briefly, the slides were deparaffinized. Antigen retrieval was done by heating in 10 mM sodium citrate buffer (pH 6.0) at 98°C, incubated in 1% hydrogen peroxide (H_2_O_2_), blocked with 5% BlokHen with avidin D, incubated with ZIP1 antibody in 5% BlokHen with biotin (Vector Laboratories) at 4°C over night followed by incubation with Horseradish peroxidase-labeled goat anti chicken IgY secondary antibody in a dilution of 1:200 (AvesLabs, Tigard, Oregon). Color was developed by incubating slides with DAB kit (Vector Laboratories) followed by Hematoxylin counterstaining. Sections were examined with light E600 Nikon microscope. Pictures were taken with Spot advanced soft ware (version. 4.0.1). The appearance of membrane-associated hZIP1 immuno-positivity of the glandular epithelial cells was used for scoring as previously described [40]. The scores employed were; negative, no positive cells; + <10% positive cells; ++ 10–50% positive cells; +++ > 50% positive cells. The mean scores between groups were analyzed by the Student's t-Test.

### 3. The ClaflinU. Study

#### RT-in situ-PCR of Human Tissue Sections

Fresh frozen sections from 38 post-prostatectomy of men with clinical histories of prostate cancer were processed for zinc content analyses and RT-in situ-PCR. RT-in situ-PCR of the frozen sections was performed as described in detail by Rishi et al [18]. To preserve the intensity of the hybridized probes, the tissues were not counter-stained. Parallel hematoxylin and eosin-stained slides were used to identify various histologic cell types in the tissue sections. Microscopic examination usually reveals cytoplasmic staining for mRNA versus nuclear staining for DNA. Cell enumeration was performed on coded slides by at least two pathologists.

#### Determination of Intracellular Zinc Content

The relative intracellular zinc content in situ was determined by utilizing fresh frozen tissues. For this purpose the cells must be biochemically active. The relative concentrations of zinc in various cell types of the prostatic tissues were determined according to the manufacturer's instructions (Molecular Probes, Inc., Eugene, Oregon, USA). Briefly, the frozen tissues were incubated with equal molar concentrations of two zinc-indicator dyes; Newport Green (NPG), and TSQ. The frozen tissues were incubated in 20 ul/section of the zinc indicator cocktail over night and washed in PBS, gently, without disturbing the tissues. The slides were heat-fixed for 10 sec at 104°C to immobilize the signals. These slides were mounted with solution containing 50% glycerol in PBS and observed under a fluorescent microscope. TSQ has a high affinity for zinc (Kd~10 nM) and a detection limit of ~0.1 nM. The ZN-TSQ positive cells stain red. NPG has moderate zinc-binding affinity (Kd ~1 μM). The ZN-NPG positive cells appear yellowish green. Together, TSQ and NPG provide a relative difference in zinc concentrations in various cell types of the prostate. TSQ has about 2–3-log higher affinity for zinc than NPG, but has detection limit of about 3-log lower than NPG. Therefore, the cells that contain very low concentrations of intracellular zinc appear red and the ones with higher concentrations appear green. The cells with no detectable Zn2+ will appear black or dark blue.

## Authors' contributions

**Umaryland Study: **RBF and LCC conceived and directed the study, wrote the Umaryland studies, wrote the final manuscript. BM performed ZIP immunohistochemical study. PF provided malignant cells and performed Western blots. **Roswell Park Study: **KS directed the study. MD obtained and conducted analyses of prostate cancer slides. KS and MD wrote the Roswell Park studies. **ClaflinU Study: **AK-D provided human tissue samples, performed histopathology, made the diagnosis and cataloged the tissues. OB performed the in situ RT-PCR on slides, developed the in situ zinc method, wrote the ClaflinU studies
